# Ultracompact single-nanowire-morphed grippers driven by vectorial Lorentz forces for dexterous robotic manipulations

**DOI:** 10.1038/s41467-023-39524-z

**Published:** 2023-06-24

**Authors:** Jiang Yan, Ying Zhang, Zongguang Liu, Junzhuan Wang, Jun Xu, Linwei Yu

**Affiliations:** School of Electronic Science and Engineering, National Laboratory of Solid-State Microstructures, Nanjing University, 210023 Nanjing, China

**Keywords:** NEMS, Mechanical engineering, Nanowires

## Abstract

Ultracompact and soft pairwise grippers, capable of swift large-amplitude multi-dimensional maneuvering, are widely needed for high-precision manipulation, assembly and treatment of microscale objects. In this work, we demonstrate the simplest construction of such robotic structures, shaped via a single-nanowire-morphing and powered by geometry-tailored Lorentz vectorial forces. This has been accomplished via a designable folding growth of ultralong and ultrathin silicon NWs into single and nested omega-ring structures, which can then be suspended upon electrode frames and coated with silver metal layer to carry a passing current along geometry-tailored pathway. Within a magnetic field, the grippers can be driven by the Lorentz forces to demonstrate swift large-amplitude maneuvers of grasping, flapping and twisting of microscale objects, as well as high-frequency or even resonant vibrations to overcome sticky van de Waals forces in microscale for a reliable releasing of carried payloads. More sophisticated and functional teamwork of mutual alignment, precise passing and selective light-emitting-diode unit testing and installation were also successfully accomplished via pairwise gripper collaborations. This single-nanowire-morphing strategy provides an ideal platform to rapidly design, construct and prototype a wide range of advanced ultracompact nanorobotic, mechanical sensing and biological manipulation functionalities.

## Abstract (web summary)

A single nanowire morphing strategy has been established to construct ultracompact soft robotic grippers, capable of large amplitude multi-dimensional maneuvering and dexterous manipulation of microscale objects, under swift Lorentz forces driving.

## Introduction

**Fig. 1:**
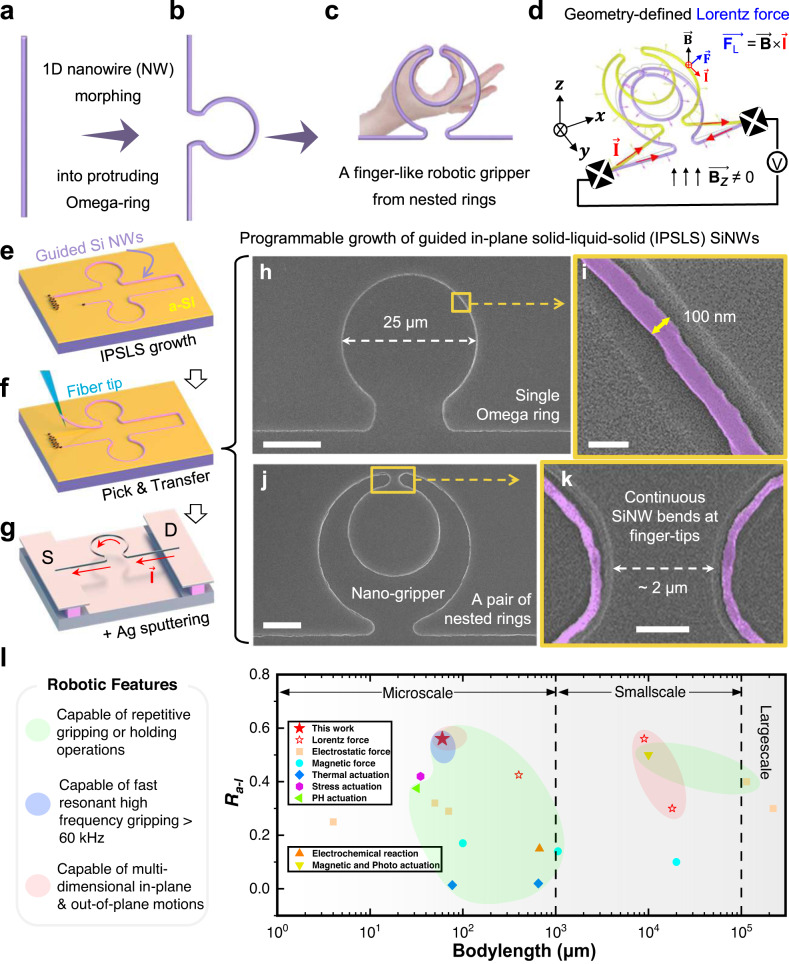
Design principle and fabrication of single-nanowire (NW)-morphed robotic grippers. **a**–**c** Illustrate the geometry morphing of a straight NW into single and nested omega-rings, as the simplest form of a pairewise gripper. **d** Schematic illustration of the opening and gripping deformations of a NW-gripper, lying on *x–y* plane, driven by geometry-defined Lorentz forces (F_L_) within a normal magnetic field in *z*-direction (**B**_*z*_). **e** The typical planar guided growth of ultralong SiNWs into designable robotic shapes, via an IPSLS mode, led by indium (In) droplets moving along the pre-defined edge lines, absorbing amorphous silicon (a-Si) precursor layer to produce crystalline SiNWs. **f**, **g** Pick-and-Place transferring of the tailored SiNWs onto a pair of bottom-isolated electrode platforms, followed by depositing a silver (Ag) capping layer to form stacked Ag/Si NWs, suspended and fixed at the two ends on the electrodes (S and D). Typical SEM images of the as-grown SiNW single and nested double omega-rings(purple) are presented in **h**–**k**. Scale bars in **h**, **i**, **j**, and **k** stand for 10 μm, 200 nm, 10 μm and 1 μm, respectively. **l** Comparison of the SiNW-morphed grippers to the micro or small-scale grippers in the literature, in terms of the ratios of motion-amplitude to body length of various (${R}_{a-l}$), while the colored & shaded areas highlight the additional motion features.

Miniaturized robotics, with high dexterity, flexibility and maneuverability, are indispensable actuators to explore novel biological, medical, opto- and electromechanical applications^1–7^. Among them, finger-like robotics of with widely varied sizes and shapes represent one of the most popular, as well as advantageous, bionic design to accomplish precise manipulation and tactical assembly^8–11^. As the most compact form of pairwise robotics, gripper can be shaped by folding an ultralong one-dimensional (1D) nanowire (NW) into a single or a pair of nested omega-rings, as depicted in Fig. 1a–c, as they are topologically identical structures. To implement this NW-morphing strategy, a prerequisite capability is to precisely control the line-shape of ultrathin 1D NWs, with diameter down to ~100 nm required for high flexibility in microscale, as well as a suitable driving scheme for achieving multi-dimensional large-amplitude motions. For example, one could conceive a suspended NW-morphed gripper structure, as depicted in Fig. 1d, which carries a passing current and thus subject to vectorial Lorentz forces (LF) of ${\vec{{{{{{\bf{F}}}}}}}}_{{{{{{\rm{L}}}}}}} \sim \vec{{{{{{\bf{B}}}}}}}\times \vec{{{{{{\bf{I}}}}}}}$, when being placed within a normal magnetic field **B**_*z*_^12^. Notably, the LF is always acting on the slim, curved NW sidewall in normal direction, which is also the most effective manner to drive the elastic NW structure into large magnitude motion, and even high-frequency resonant vibrations.

Compared to the other driving schemes of robotic actuators, as summarized in Supplementary Table 1, with two or more movable arms driven by electrostatic attraction^13–18^, magnetic field^19–24^, thermal expansion^25–27^, chemical reaction^28,29^, illumination^30^, pressurized voids^31^, residual stress^32^, or protein-muscle stimulation^33^, the combination of flexible NW-morphing and LF-driving exempts the need of approached electrodes^34–36^, and can be swifter, easier-to-control and more adaptive to multi-dimensional large motions. Particularly, the ratio of the motion-amplitude over the body-length of grippers (*R*_*a-l*_) has been established as an important figure-of-merit that measures the effectiveness or potential maneuverability of the gripper actuators in a miniaturized working environment^37^. As summarized in Fig. 1l, there remains still a strong need for the development of ultracompact ( < $100\,{{{{{\rm{\mu }}}}}}{{{{{\rm{m}}}}}}$) and high *R*_*a-l*_ grippers, which can better fit into smaller working field for high-precision manipulation. To this end, a single-NW-morphed gripper represents probably the simplest and the most compact LF-driven robotics, but this has never been explored so far.

In this work, we propose an ultracompact design of robotic grippers, which are composed of nested omega-ring structures constructed via continuous NW-morphing of an ultralong and thin Si NWs grown via an in-plane solid-liquid-solid (IPSLS)^38–49^ mechanism. Via a reliable transferring and suspended upon electrode platforms, these NW grippers demonstrate an extraordinarily high flexibility that allow swift LF-driving to accomplish a wide range of agile large-amplitude 3D maneuvers, a series of dexterous robotic manipulations and reliable releasing of microspheres and microrods. Further on, more advanced double-hand collaborations and on-site LED unit picking and lighting are also demonstrated for the first time. This NW-morphing and LF-driving strategy will provide a whole new and powerful platform to rapidly design, fabricate and testify a rich set of bionic microscale robotic structures and functionalities.

## Results

### SiNW growth and geometry design

The ultralong SiNWs, with typical length ${L}_{{{{{{\rm{nw}}}}}}} > 200\,{{{{{\rm{\mu }}}}}}{{{{{\rm{m}}}}}}$ and diameters of ${D}_{{{{{{\rm{nw}}}}}}} \sim 150\pm 20\ {nm}$, were grown via an IPSLS mechanism established in our previous works^46–49^, by using indium (In) droplets as catalyst that absorb pre-coated amorphous Si (a-Si) layer precursor to produce crystalline SiNWs. During this course, the In droplets can be attracted and guided by the pre-patterned step edges to produce continuous SiNWs with designable layout, as depicted schematically in Fig. 1e. More experimental details of the IPSLS growth of SiNWs are available in the Material and Method session or previous works^38–49^. Tailored SiNWs of single or double-nested omega rings were first grown upon patterned SiO_2_/wafer substrate, as seen for example in the scanning electron microscopy (SEM) images presented in Fig. 1h–k. As a key basis for the construction of NW-morphed grippers, the guided growth of such ultralong SiNWs can be rather stable and continuous, even over the sharply turning tracks with a local radius of curvature (ROC) < 1.5 μm.

The as-grown SiNW rings were first picked up by using sharp fiber tips, as depicted in Fig. 1f and explained more in Methods Section, prior to be placed upon a pair of bottom-isolated electrodes separated ${L}_{{{{{{\rm{span}}}}}}}$ ~ 70 ${{{{{\rm{\mu }}}}}}{{{{{\rm{m}}}}}}$ apart (Fig. 1g). Then, a thin layer of 80 nm silver (Ag) thick was evaporated by using magnetron sputtering over to form a highly conductive Ag/SiNW pathway (with resistance of ~400 Ω) that connects the two platform electrodes. Thanks to a concave belt at the roots of the platform, as shown in Supplementary Fig. 1, the top surfaces of the two platforms are isolated from the ground surface. Within a normal magnetic field of$\,\vec{{{{{{{\bf{B}}}}}}}_{{{{{{\rm{z}}}}}}}}$, the suspended Ag/SiNW will experience vectorial LF, when passing a current flow under a bias of ${V}_{{{{{{\rm{bias}}}}}}}$, acting on the slim NWs with the same magnitude but in perpendicular direction to the sidewalls. Note that, the LF exerting on the flexible NWs can be swiftly controlled by simply tuning the electric bias or the passing current.

**Fig. 2:**
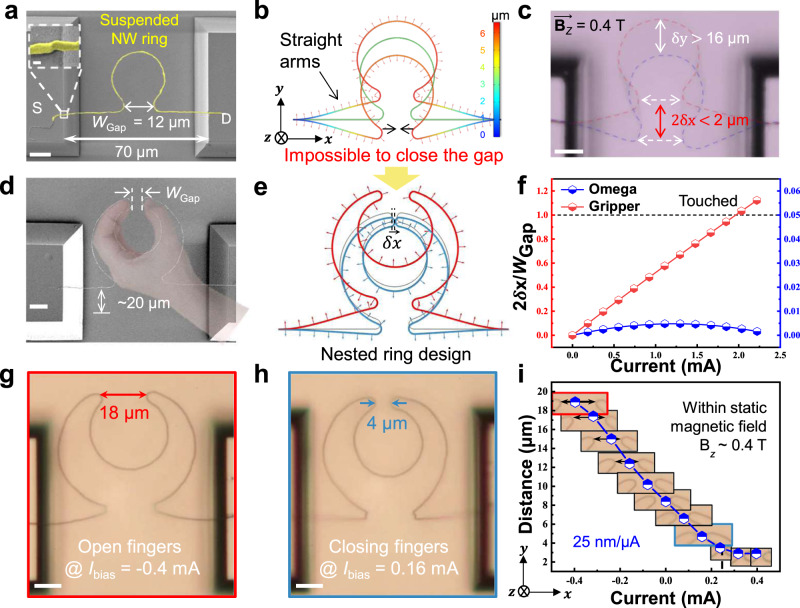
Structural design for grippers. **a** SEM image of a suspended Ag/SiNW omega ring(yellow, the tip-separation gap ${W}_{{{{{{\rm{gap}}}}}}}=12\,{{{{{\rm{\mu }}}}}}{{{{{\rm{m}}}}}}$), spanning ${L}_{{{{{{\rm{span}}}}}}}=\,$70 ${{{{{\rm{\mu }}}}}}{{{{{\rm{m}}}}}}$ over two electrodes (S and D) plateaus, which can be easily actuated to vibrate largely in *y*-direction but unable to achieve the desired gripping functionality, driven by the LF under alternating current ${I}_{{{{{{\rm{bias}}}}}}}={I}_{0}\cos \omega t$ within **B**_*z*_ = 0.4 T, while the simulated displacement/force fields and real-time microscope image of a ring at resonant vibration @$\omega=80\ {{{{{\rm{kHz}}}}}}$ are shown in **b** and **c**, respectively. In contrast, a nested double-ring configuration, as seen in **d**, **e**, forms the simplest bionic gripper, which enjoys a much larger displacement freedom in *x*-direction, $\delta x$. **f** Comparison of the tip-to-tip separations achieved by the single and nested double rings, under the same driving current and magnetic field. **g**, **h** present the images of the Opening and Closing states of the gripper, recorded at constant electric biases of *I*_bias_ = −0.4 mA and 0.16 mA, respectively, while the detailed tip-to-tip separation and snap-images under different bias current are extracted and plotted in **i**. Scale bars, 10 μm for a, c, d and g, 9 μm for h.

As seen in Fig. 2a, b, with ${{{{{{\bf{B}}}}}}}_{z}=0.4\ {{{{{\rm{T}}}}}}$ and passing current of $\left|{\widetilde{I}}_{{{{{{\rm{bias}}}}}}}\right |=0.5\ {{{{{\rm{mA}}}}}}$, the suspended single Omega-ring can be actuated into large-amplitude vibration in *y*-direction, by a LF with a magnitude estimated to be ${{{{{\rm{|}}}}}}{\mathop{{{{{{\bf{F}}}}}}}\limits^{ \rightharpoonup }}_{y} |={{{{{\rm{|}}}}}}{\mathop{{{{{{\bf{B}}}}}}}\limits^{ \rightharpoonup }}_{z}\times {\mathop{{{{{{\bf{I}}}}}}}\limits^{ \rightharpoonup }}_{{{{{{\rm{ring}}}}}}}{L}_{{{{{{\rm{span}}}}}}}{{{{{\rm{|}}}}}} \sim 3\,{{{{{\rm{\mu }}}}}}{{{{{\rm{N}}}}}}$. By tuning the driving frequency to ${f}_{{{{{{\rm{res}}}}}}}=80\,{{{{{\rm{kHz}}}}}}$ (Fig. 2c), the suspended Omega-ring is excited to oscillate resonantly with large amplitude in *y*-direction of ${\delta }_{y} \sim 16\,{{{{{\rm{\mu }}}}}}{{{{{\rm{m}}}}}}$, see more details in Supplementary Fig. 2 and Supplementary Movie 1. However, this single omega-ring is fundamentally constrained by the two straight arms, which will not allow them to touch for a holding gesture. To relax this constraint, a nested double-ring or gripper has been conceived and fabricated, as shown in Fig. 2d, e, which under the collaborative LF exerted on the inner and outer rings, but in opposite directions due to the inverse current circulating direction, the two fingertips can easily touch each other. The advantage of such more flexible nested-omega-ring structure, to accomplish a large *x*-axis displacement for holding, can also be seen from the simulated change of the gap-closing ratio, defined as ${{{{{\rm{R}}}}}}\equiv 2{\delta }_{x}/{W}_{{{{{{\rm{gap}}}}}}}$, under different driving current, as presented in Fig. 2f, which is a key capability for the gripper to really hold or pick up micro objects for further manipulation.

Indeed, the Close (hold) and Open (release) states of the grippers can be clearly observed in experiments, as presented in Fig. 2g, h, achieved under *I*_bias_ = −0.4 mA and 0.16 mA, respectively, while the tip-separation gap ${W}_{{{{{{\rm{gap}}}}}}}$ is continuously tunable by convenient current control. As witnessed in Fig. 2i, a series of photo snapshots are shown at different bias currents, revealing a quasi-linear response in the bias range of $-0.4\ {{{{{\rm{ mA}}}}}} \, < \,{I}_{{{{{{\rm{bias}}}}}}} \, < \,0.2\ {{{{{\rm{ mA}}}}}}$, while for even larger bias, the finger-tip separation seems to saturate at a minimum gap of ${W}_{{{{{{\rm{gap}}}}}}}^{{{\min }}} \sim 2.8\,{{{{{\rm{\mu }}}}}}{{{{{\rm{m}}}}}}$ for this specific gripper. It is important to note that, the size/dimension design of the gripper has been optimized to achieve simultaneously a large enough displacement for pairwise gripping, and a reasonable structural stability against undesired deformation. For example, for the given diameter of NW, further down-scaling the size of the gripper will reduce its flexibility or maneuverability (as witnessed in Supplementary Fig. 3a), while a too large design of the gripper will make it softer under the same current LF-driving, but prone to deform under gravity dragging or become unstable under contractive LF-driving to hold, as seen in Supplementary Fig. 4, that the inner ring will opt to incline out of the *x–y* operating plane, to release the accumulated LF stress, but hinders further closing of the tips. Also, during a fiber-tip-assisted transferring and assembly process, the slim SiNW segments of the inner and outer rings, with the largest inner ring design (for example ${D}_{{{{{{\rm{rings}}}}}}}^{{{{{{\rm{in}}}}}}/{{{{{\rm{out}}}}}}}=32$ ${{{{{\rm{\mu }}}}}}{{{{{\rm{m}}}}}}$/40 ${{{{{\rm{\mu }}}}}}{{{{{\rm{m}}}}}}$, see Supplementary Fig. 3b), are too close to each other and thus easy to mingle together, causing undesired distortions and LF control complexity. So, for the SiNW of ${D}_{{{{{{\rm{nw}}}}}}} \sim 150\ {{{{{\rm{nm}}}}}}$, the adopted inner/outer ring diameters of ${D}_{{{{{{\rm{rings}}}}}}}^{{{{{{\rm{in}}}}}}/{{{{{\rm{out}}}}}}}=$24 ${{{{{\rm{\mu }}}}}}{{{{{\rm{m}}}}}}$/40 ${{{{{\rm{\mu }}}}}}{{{{{\rm{m}}}}}}$ represents a good trade-off to achieve both a large motion-amplitude and a stable gripper structure for robotic operations.

### Robotic picking and multi-dimensional manipulation

**Fig. 3:**
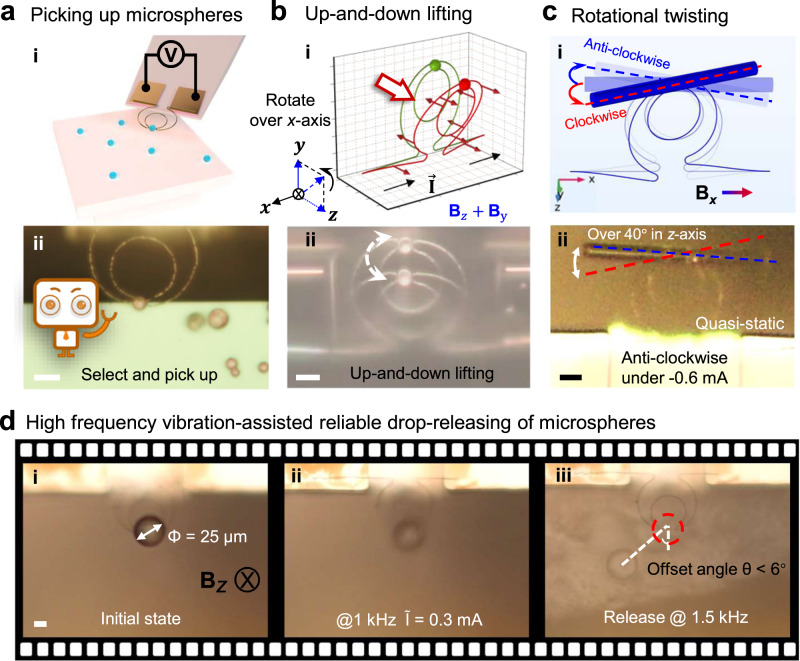
Robotic picking, multi-dimensional manipulation and releasing. **a** Illustrates the goal of selective capturing and transferring of microscale objects. **b** Up-down lifting in *z*-direction by rotating into effective **B**_*y*_ = 0.4 T, while a Lorentz force vector analysis of this maneuver is provided in **(ii). c** Quasi-static twisting over $\pm \,{20}^{{{{{{\rm{o}}}}}}}$ under constant bias currents and **B**_*X*=_0.4 T. **d** High-frequency vibration assisted release of a microsphere on gripper under magnetic field along *z*-direction at 1.5 kHz. $\widetilde{I}:\,$Alternating current bias. Scale bars all stand for 10 ${{{{{\rm{\mu }}}}}}{{{{{\rm{m}}}}}}$.

In view of serving as potential manipulators for NEMS sensing and biological diagnostic or treatments, the grippers are supposed to capture tiny inorganic objects or cells with typical size of 4–9 ${{{{{\rm{\mu }}}}}}{{{{{\rm{m}}}}}}$^2,32,33^. Here, SiO_2_ microspheres of $\sim 4\,{{{{{\rm{\mu }}}}}}{{{{{\rm{m}}}}}}$ in diameter, as diagrammed and seen under microscope in Fig. 3a, are taken as the model targets, which were first spread over the surface of SiO_2_/wafer substrate, as a mining place for the gripper to selectively pick up for subsequent operation. The gripper was mounted upon the tip of a manually controlled stage, with two electrodes connected to pass current (${I}_{{{{{{\rm{bias}}}}}}}$) to impose LF-driven robotic motions. Under a background magnetic field of ${{{{{{\bf{B}}}}}}}_{z}=0.4\ {{{{{\rm{T}}}}}}$ and a bias of ${I}_{{{{{{\rm{bias}}}}}}}=-0.16\ {{{{{\rm{mA}}}}}}$, the tip gap of the gripper can be opened to 12 ${{{{{\rm{\mu }}}}}}{{{{{\rm{m}}}}}}$ to fit in and pinch a microsphere, and then held it, by setting ${{{{{{\rm{I}}}}}}}_{{{{{{\rm{bias}}}}}}}=0\ {{{{{\rm{mA}}}}}}$, and retracted it back from the substrate, against the van der Waals (vdW) force between the microsphere/substrate, by navigating the holding stage. It is noteworthy that the picking of the 4 ${{{{{\rm{\mu }}}}}}{{{{{\rm{m}}}}}}$-sized microspheres is very reliable, with 100% successful rate, and takes only <20 s for the experienced operators. The detail of microsphere picking from the edge of a platform by a LF-driven gripper was provided in Supplementary Movie 2. Note that, the tip-separation for this specific gripper is designed to be slightly smaller than the diameter of the microsphere, so that the elastic tips will clip the sphere when no LF was applied, that is under zero bias. However, with this tip-separation of 8 μm, grabbing a much smaller microsphere, say diameter <1 μm, was found to be difficult, largely due to the missing of natural elastic clipping mechanism and the difficulty for microscope observation. Of course, this is readily adjustable as the tip separation is only subject to the conventional photolithography resolution ( $\sim$ $2\,{{{{{\rm{\mu }}}}}}{{{{{\rm{m}}}}}}$) used in this work. On the other hand, for gripping conductive objects, the gripper can be pre-coated with an insulating layer of Al_2_O_3_ or SiN_*x*_ to prevent the shorting of passing current at the touched tips, which provides also an extra means to modify the surface chemistry, adhesion and bio compatibility of the gripper sidewalls.

In parallel, multi-dimensional out-of-plane lifting and twisting operations are also highly valuable capabilities in practical robotic manipulations. By gradually turning the grippers around the *x*-axis, which is equivalent to switching the applied magnetic field from ${\mathop{{{{{{\bf{B}}}}}}}\limits^{ \rightharpoonup }}_{z}$ to ${\mathop{{{{{{\bf{B}}}}}}}\limits^{ \rightharpoonup }}_{y}$, the grippers can be actuated to flap swiftly in the *y–z* plane, as indicated and shown in Fig. 3b and Supplementary Fig. 5, to lift the carried microsphere up-and-down by simply reversing the current flow direction vertical *y-*direction. According to the force analysis in Fig. 3b(i), during a flapping maneuver, though the inner and the outer rings are torqued to opposite directions, along the *x*-axis, causing thus a small intersection angle between the two ring planes, this will not influence much the holding and flapping operations with the optimal size design as mentioned above. Note that, this out-of-plane (gripper lying *x–y* plane) flapping or lifting capability is rather useful for the gripper to place/detach micro-payload onto/from counter platforms.

Furthermore, local twisting maneuver can be excited by applying a lateral magnetic field in *x-*direction, as illustrated schematically in Fig. 3c. The fiber segment was first attached to two arms of the gripper, stuck basically by the vdW force, then a series of electric-controlled twisting maneuvers were exerted within a constant magnetic field of **B**_*x*_ = 0.4 T. Due to the much larger contact area between the long fiber segment and the arms of the gripper, this vdW force can be strong enough to hold the fiber firmly even during the twisting operation. Interestingly, the fiber segment can be quickly twisted back and forth, over a quasi-static deviation angle up to $\pm {20}^{{{{{{\rm{o}}}}}}}$, under a ${{{{{\rm{|}}}}}}\widetilde{I}{{{{{\rm{|}}}}}}$ = 0.6 mA. This twisting behavior can be precisely controlled by electrical biases in microscale and operate swiftly in high frequency and even at resonant mode, to achieve a much larger vibration amplitude, for example as witnessed in Supplementary Fig. 6 and Supplementary Movie 3, where the first/lowest resonant frequency of 4.4 kHz was identified. Note that, this local twisting capability, without the need to rotate the axis of the holding platform or electrodes is particularly suited for convenient manipulations of tiny bio-objects, to capture and adjust them into precise alignments, ready for further testing or treatment. This capability is indeed widely needed, for instance, to hold and rotate single oocyte cell to achieve higher enucleation success rate or better understand the characteristics of living cells^24,50–52^. As for clamping impact on the living cells, exerted by the gripper at the tips, which is indeed adjustable by tuning the equilibrium tip-gap, or can be estimated via FEA simulation (Supplementary Fig. 7), which indicates that the clipping pressure at the contacts should be in the range of 8.6 to 54 kPa, under bias current of 0-4 mA, which is soft enough to gently hold the cells without the risk of penetrating through the cell membranes^53,54^.

### High-frequency vibration assisted releasing

In addition to the picking and manipulation, there is still another challenging issue encountered for the delivery of microscale payload, arising from the sticky vdW force that becomes increasingly prevailing in microscale^55,56^ and usually prevents an easy release of tiny object onto targeted places. To achieve a straightforward and accurate releasing at desired place, the LF-driven vibration of the gripper has an outstanding advantage. This is derived from the fact that a high-frequency small amplitude vibration at the holding tips can help to quickly diminish and break the local vdW adhesive force to separate micro-payload from the carrier’s surface. As a straightforward demonstration of this unique capability, Fig. 3d(i-iii) shows the releasing process of a large microsphere with diameter of $\sim 25\,{{{{{\rm{\mu }}}}}}{{{{{\rm{m}}}}}}$ (for the ease of observation) from the gripper. With a background magnetic field of 0.4 T, the gripper lying in *x–y* plane was passed with an alternating current of 0.3 mA and activated to a kind of tip open-close vibration, as showcased in Fig. 2. It was found that, the microsphere can stick firmly to the gripper tips when the actuation frequency increased from 0 to 1 kHz, but fell basically vertically to ground surface at a higher frequency at 1.5 kHz, with an offset angle of <6 Degree. More dynamic details are provided in Supplementary Movie 4. During all this actuation-releasing process, the mass center position of the microsphere remained almost unchanged, allowing thus a first alignment and then vertical dropping of microsphere. Note that, this electrically controlled vibration disturbance and be applied superimposing upon a background bias signal, and has proven rather helpful for achieving a precise placing of tiny objects onto the desired locations in this work.

### Elastic deformation and stability under large dragging

**Fig. 4:**
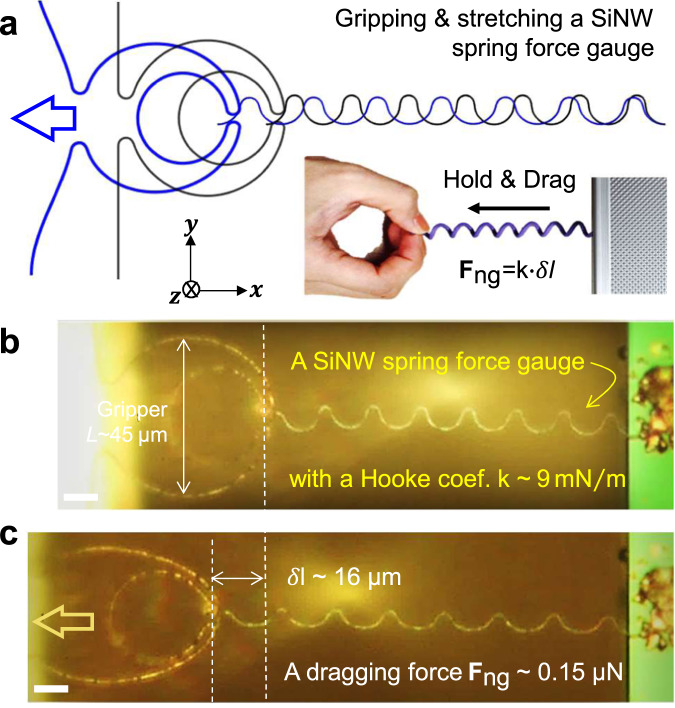
Elastic deformation, stability and force gauging of the NW gripper. **a** Diagram of a gripper holding the end of a SiNW spring, serving as a force gauge to estimate the dragging force (F_ng_) exerted by the pulling gripper. **b**, **c** The photos of the initial and the stretched states of a gripper pulling a spring gauge, respectively. Scale bars all stand for 10 μm.

In order to assess its overall elastic deformation or adaptability, the gripper was also employed to pick up a slim SiNW spring, as a delicate force gauge, to measure the steadiness of the imposed holding and pulling strength. As shown in Fig. 4a, b, the spring gauge was grown via single-run of IPSLS process, with an average diameter of 100 nm and an elastic meandering line-shape for extra stretchability, and fixed at one end by silver epoxy on a platform edge and gripped at another end by the gripper. According to the SEM observations (Supplementary Figs. 8a, b) and the finite element simulation (Fig. 4a), this specific SiNW spring is estimated to have an elastic Hooke coefficient of $k\ \sim\ 9\ {{{{{\rm{mN}}}}}}/{{{{{\rm{m}}}}}}$. Thus, under a bias of ${I}_{{{{{{\rm{bias}}}}}}}\,=\,$0.5 mA to grip firmly the left end of the spring, the effective dragging force can be estimated from the elongation of the elastic spring, of ${\delta }{l} \sim 16\,{{{{{\rm{\mu }}}}}}{{{{{\rm{m}}}}}}$, by comparing its initial and stretched states in Fig. 4b, c, which corresponds to a pulling force of ${{{{{{\bf{F}}}}}}}_{{{{{{\rm{ng}}}}}}} \sim 0.15\,{{{{{\rm{\mu }}}}}}{{{{{\rm{N}}}}}}$ exerted by the gripper. In response, the gripper by itself was deformed and shrunk to 85 % of the initial width, measured in vertical direction, as seen in Fig. 4c. Further pulling leads to a sudden detaching of the spring end from the holding tips, which could result from an increased counter-force of the spring, as well as the fact that such a stretching-caused deformation will enforce the gripper to open-up or loose the holding of the spring tip, thus reducing the local effective gripping force, which is also an important factor that need to be taken into account in functional design and practical implementation.

Also, during this extremal force-gauging process, lasted for several seconds under bias current of ~0.5 mA, the Joule heating power is estimated to be ${P}_{{{{{{\rm{Joule}}}}}}} \sim \,{I}^{2}R=50\,{{{{{\rm{\mu }}}}}}{{{{{\rm{W}}}}}}$, which is estimated (not measured) to cause a local temperature increase of $\Delta T \sim$ 90 ^o^C in the suspended Ag-coated SiNW grippers, considering only the heat dissipation through the two holding arms. Actually, under this driving condition, it has been experimentally observed that the gripper can work stably for more than 1 h in ambient condition. However, special caution should be taken to avoid the use of a too large driving current for too long, for example >2 mA for >2 min, as the temperature could quickly soar up to fuse the conductive Ag layer, and even break the suspended SiNW framework. Despite of the potential heating damage that could be caused, by LF-driving in vacuum and at extremal situations, most of the gripping behaviors can be operated safely at much lower power regime.

### Collaborative and pairwise teamwork manipulations

**Fig. 5:**
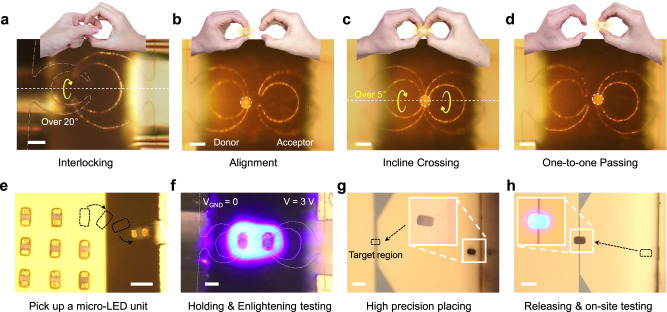
Collaborative hand-to-hand passing of microsphere and micro-LED pixel repairing. **a** Sophisticated double-hand collaboration demonstrated by a pair of robotic grippers. **b**–**d** A precise hand-to-hand passing of a microsphere from the left to the right robotic arms. **e** Picking up a micro-LED from substrate by a LF-driven gripper. **f** Micro-LED was lightened in suspension by applying a bias voltage between the two grippers to ${V}_{{{{{{\rm{drive}}}}}}}=3\,{{{{{\rm{V}}}}}}$. **g**, **h** Micro-LED unit was transport to the target location and enlightened by the local electrode pair. Scale bars in a-d and f stand for 10 ${{{{{\rm{\mu }}}}}}{{{{{\rm{m}}}}}}$, and in e, g and h stand for 50 ${{{{{\rm{\mu }}}}}}{{{{{\rm{m}}}}}}$.

A pair of grippers can accomplish more advanced teamwork collaborations, for example the posing of interlocking gesture with a slight intersect angle, to be ready for passing a clipped microsphere into the hand of another gripper (Fig. 5a). Specifically, a collaborative passing of the held object, from a donor gripper to the acceptor counterpart, was demonstrated in Fig. 5b–d, where the acceptor one on the right was first tuned to have a slight incline angle of ~5 Degree, and then navigated to approach the donner tip. After applying a LF-driven holding (by passing current) at the acceptor gripper, and a releasing (stop current) at the donner one, the gripping of the microsphere load was passed safely to the acceptor gripper. This kind of double-hand collaboration, enabled by the bionic design and the flexible holding gesture, is supposed to be useful for establishing a reliable inter-gripper transfer protocol, which will be needed to link the different stages with varied functionalities, such as object acquisition, dispatching and subsequent treatment and analysis.

As another example of the collaborative gripper functionalities, a pair of grippers were exploited to selectively pick up, on-site examine and precisely install micro light-emitting diode (micro-${{{{{\rm{LED}}}}}}$). The micro ${{{{{\rm{LED}}}}}}$ units, of 20$\,{{{{{\rm{\mu }}}}}}{{{{{\rm{m}}}}}}$ wide and 35$\,{{{{{\rm{\mu }}}}}}{{{{{\rm{m}}}}}}$ long, were fabricated and packed as orderly array on wafer Substrate (Fig. 5e), with coplanar electrodes exposed on the top facet. A gripper was navigated to pick up one unit, by clamping the gripper tips over the protruded Au electrodes, dragging it away from parent substrate. Then, another gripper was manipulated to approach and hold the other electrode pads of the suspended micro-LED unit. By applying a bias voltage between the two grippers to ${V}_{{{{{{\rm{drive}}}}}}}=3{{{{{\rm{V}}}}}}$, the micro-LED can be directly testified and lightened, all in suspension as witnessed in Fig. 5f, thanks to the conductive finger tips and the excellent clamping contacts to the electrodes. After that, this micro-LED unit can be transported to be above the gap of a pair of planar electrodes, as marked in Fig. 5g, h, by the right gripper, and then flipped over to be installed, in a face-down manner, to ensure a perfect contact between unit pads to the ground electrodes. Finally, the installed micro-LED was successfully enlightened on the desired place, powered by the local electrode pair, as seen in the inset of Fig. 5h. This micro-LED manipulation and lightening operation provides a good example that the grippers can be dexterous enough for multifunctional tasks that demand a high level of maneuverability and flexibility, and suitable for handling complex missions of microscale transport, 3D manipulation and precise installation, thanks to the pairwise geometry design and vectorial LF-driving scheme.

## Discussion

In comparison to the other bionic grippers reported in the literature, as summarized in Supplementary Table 1, this SiNW-morphed ultracompact grippers can accomplish (1) a rich set of pairwise robotic manipulations, with feature dimensions much smaller than most of the gripper robotics, while single NW-morphing strategy represents the simplest topological structure to compose a gripper suitable for the implementation of LF-driving scheme; (2) As highlighted in Fig. 1l, this small gripper fabricated out of a single SiNW wiring represents the smallest version of robotics that can achieve the largest motion-amplitude to body-length ratio, approaching ${R}_{a-l}$ > 0.6; (3) These slim, soft and highly elastic grippers can be driven swiftly to open and close the tip gap, in ${\tau }_{{{{{{\rm{op}}}}}}}$ < 3 μs under a convenient electric current ($\left|{I}_{{{{{{\rm{bias}}}}}}}\right|\, <$ 0.5 mA) control, particularly when operated in a resonant oscillation mode, as shown in Supplementary Figs. 2c, d, at a high resonant frequency >60 kHz, which is a unique capability as demonstrated in Fig. 3d, to achieve a straightforward and easy releasing of tiny object loads against the sticky vdW force or adhesion; (4) A full set of dexterous robotic behaviors and more advanced collaborative teamwork manipulations, testing and installation have been demonstrated for the first time in microscale. All these new features and capabilities are desirable for the developing a new generation of multifunctional nanorobotic units to work reliably and flexibly in ambient, liquid and even harsh high temperature and vacuum environments.

In summary, we have proposed and demonstrated a new NW-morphing strategy to construct diverse and highly maneuverable gripper robotic structures, which can be very efficiently actuated into large-amplitude motions, driven by NW-geometry-defined Lorentz forces, when carrying a tunable current flow within a background magnetic field. A rich set of dexterous bionic behaviors of grasping, flapping and twisting operations have been demonstrated, enabling a precise and multi-dimensional delivery, manipulation and releasing of various microscale sphere, rods and springs, as well as well-concerted double-hand collaborations and functional electrical testing and installation of micro-LED units. These results highlight a unique opportunity to batch-manufacture an army of ultracompact and diverse nanorobots, catering to the emerging needs of future biosensing, nanomanipulation and electro/optomechanical applications.

## Methods

### Designable IPSLS growth of ultralong and slim SiNWs

The SiNWs were grown upon SiO_2_-coated Si wafers substrates via an IPSLS growth mode as described in our previous works^38–42^. First, guiding step edges with pre-designed Omega and nested structures were defined by photolithography and subsequent etching into the underlying SiO_2_ layer to a depth of 120 nm by using inductively coupled plasma (ICP). Second, indium (In) stripes of 35 nm thick were deposited at the starting ends of the guiding edges by electron beam evaporation (EBE). The samples were then loaded into a plasma enhanced chemical vapor deposition (PECVD) system, and treated with a H_2_ plasma at 250 °C for 5 min, with gas flow rate and chamber pressure of 15 SCCM and 140 Pa, respectively. Then, an a-Si thin film of 30 nm thick was deposited at 100 °C (below the melting point of the In catalyst), with 5 SCCM pure SiH_4_ plasma and 20 Pa chamber pressure. Third, the substrate temperature was raised to 350 °C and annealed in vacuum for 60 min. During this course, the In droplets started to move and absorb a-Si thin film to produce crystalline SiNWs behind. Finally, at the end of the SiNW growth, the remnant a-Si layer was selectively etched off by using H_2_ plasma at 120 °C.

### Electrode platform formation

The paired electrode platforms, edge-to-edge separated 70 μm apart, were etched upon a silicon-on-insulator (SOI, box layer thickness of 2 ${{{{{\rm{\mu }}}}}}{{{{{\rm{m}}}}}}$) substrate by photolithography and etched by ICP with SF_6_ plasma, where the top electrode layers are isolated from the bottom wafer by the oxide layer. In order to form a concave belt at the root of and surrounding the electrode platform, the bottom exposed oxide sidewall was further etched by a 4%-diluted HF solution dipping to recede roughly ~2 μm inwards. Thanks to this special design, the inner sidewall of the concave belt would not be covered by the Ag layer during the metal deposition, and thus provide a good electric isolation of the top platform electrodes from the bottom surface. In the meantime, the suspended SiNW-ring in between will receive the Ag capping layer to form a continuous and highly conductive pathway that bridges the two electrodes.

### Assembling of NW-ring complexes

An optical fiber, with 200 nm diameter at the tip, was fixed on manually controlled XYZ manipulator and used to pick up the as-grown SiNWs from the parent wafer substrates. Then, the SiNWs were transferred and placed onto the paired electrode platforms, formed by etching into the SOI substrate, to obtain a doubly clamped suspended NW robotic structure. The subsequent sputtering coating of 80 nm Ag also helped to fix the transferred NW-ring onto the platform top surface.

### Lorentz force driving and actuations

A current flow (*I*) was applied through the suspended Ag/Si NW, within a static background magnetic field vector ($\vec{{{{{{\bf{B}}}}}}}$) with a fixed magnetic flux density of 0.4 T but adjustable direction. The current flow was controlled by a signal generator (Keysight 3390 and Keithley 2636B) that can provide pulse or sinusoidal bias voltage. Lorentz force **F**, perpendicular to the sidewall of Ag/NW, confining the current flow in 3D space, is thus caused to drive the suspended NW-ring structure into large displacement and vibrations. The vibration and deformation of the NW-rings or robots were observed and captured under optical microscopy.

### Mechanical force gauging and robotic manipulation

The pulling strength of the gripper was testified by using the SiNW spring as a force gauge. The SiNW spring, also grown via an IPSLS growth mode, was fixed by silver paste at one end upon the stage edge. The single robotic gripper on substrate approached to NW spring and picked up the spring by applying the Lorentz force. The dragging force exerted by the robotic hand was calculated by the elongation of the elastic spring. The pulling and squeezing states of a SiNW spring were manipulated by a single-hand gripper. Furthermore, the advanced double-hand teamwork in 3D space, including the interlocking gesture for object exchange, the carrying and multidirectional stretching, and even the arm-force bending of the tiny spring loads can be achieved by a pair of grippers. Note that, the dielectric coating on the gripper can be used to prevent a short-circuit at the ends that will otherwise re-route the current and LF.

### 3D microsphere handling and passing

A microsphere of ~4 μm in diameter was first loaded to the gripper by a fiber tip. The gripper could catch the microsphere firmly under positive bias current of 0.3 mA, and then the fiber tip retracted. After that, the microsphere can be translated within the *x*–*y* plane with the normal **B**_*z*_ magnetic field under bias current of 0.1 mA, or elevated up-and-down vertically by gradually turning the grippers around the *x*-axis. In addition, the reliable passing or sharing of the target micro object can be realized between the two grippers. Briefly, the donor and the acceptor grippers were first faced to each other and adjusted to lie on two planes with a slight incline angle of ~5 degree. Then, the acceptor gripper approaches and intersects with the donner at the tip region, followed sequentially by the LF-holding for the acceptor gripper and the LF-releasing by the donner one.

### Finite element simulation and mechanical analysis

The Lorentz force analysis and mechanical deformation simulation of the NW-ring complexes were carried out by using COMSOL Mechanical & Current-Voltage toolkits. The geometry dimensions, such as diameter and line-shape, were extracted from direct SEM observations. The material properties of the composite Ag/NW structures are obtained from the COMSOL Library, while the SiNW is assumed to be isotropic poly-Si with a Young’s modulus and Poisson’s ratio of 160 GP and 0.22, respectively.

## Supplementary information

Supplementary information

Description of Additional Supplementary Files

Supplementary Movie 1

Supplementary Movie 2

Supplementary Movie 3

Supplementary Movie 4

## Data Availability

All data are available in the main text or the supplementary materials.
