# Supplementary material for: Ultracompact single-nanowire-morphed grippers driven by vectorial Lorentz forces for dexterous robotic manipulations
Source: Nat Commun. 2023 Jun 24;14:3786. doi: 10.1038/s41467-023-39524-z (PMC10290722; doi:10.1038/s41467-023-39524-z)
Supplement: Supplementary file 1 — Supplementary information [file 41467_2023_39524_MOESM1_ESM.pdf]

Supplementary information for

Ultracompact single-nanowire-morphed grippers driven by vectorial Lorentz forces for dexterous robotic manipulations

Jiang Yan, Ying Zhang, Zongguang Liu, Junzhuan Wang, Jun Xu, Linwei Yu**

School of Electronic Science and Engineering, National Laboratory of Solid-State Microstructures, Nanjing University, 210023 Nanjing, China

*Corresponding authors: liuzongguang@nju.edu.cn, yulinwei@nju.edu.cn

This PDF file includes:

Supplementary Figs. 1-8

Supplementary Table 1

Supplementary References

Supplementary Figures

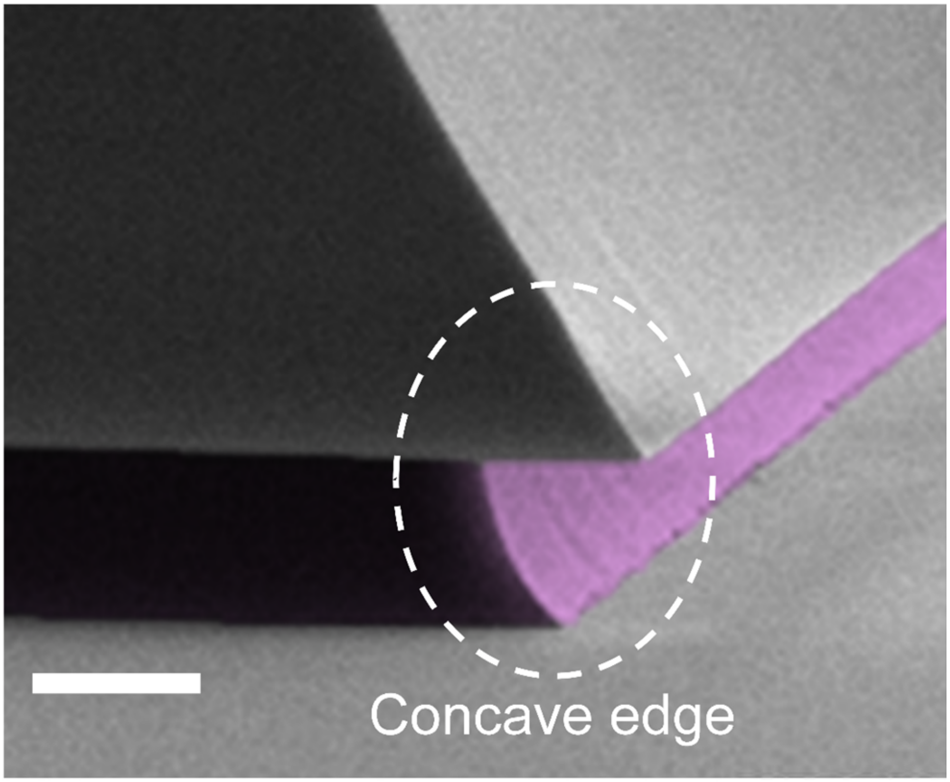

Supplementary Fig. 1 Concave bottom edge design at the roots of the electrode platform. The paired electrode platforms were first fabricated upon a silicon-on-insulator substrate by photolithography and etched by ICP with SF_6 plasma. Then, the bottom exposed oxide sidewall was further etched by a 4%-diluted HF solution to recede roughly ~2 μm inwards to form a concave belt at the root along the platform edges. In this way, the platform top surfaces can be well isolated from the ground, after the evaporation of silver layer via sputtering. The scale bar is for 2 μm.

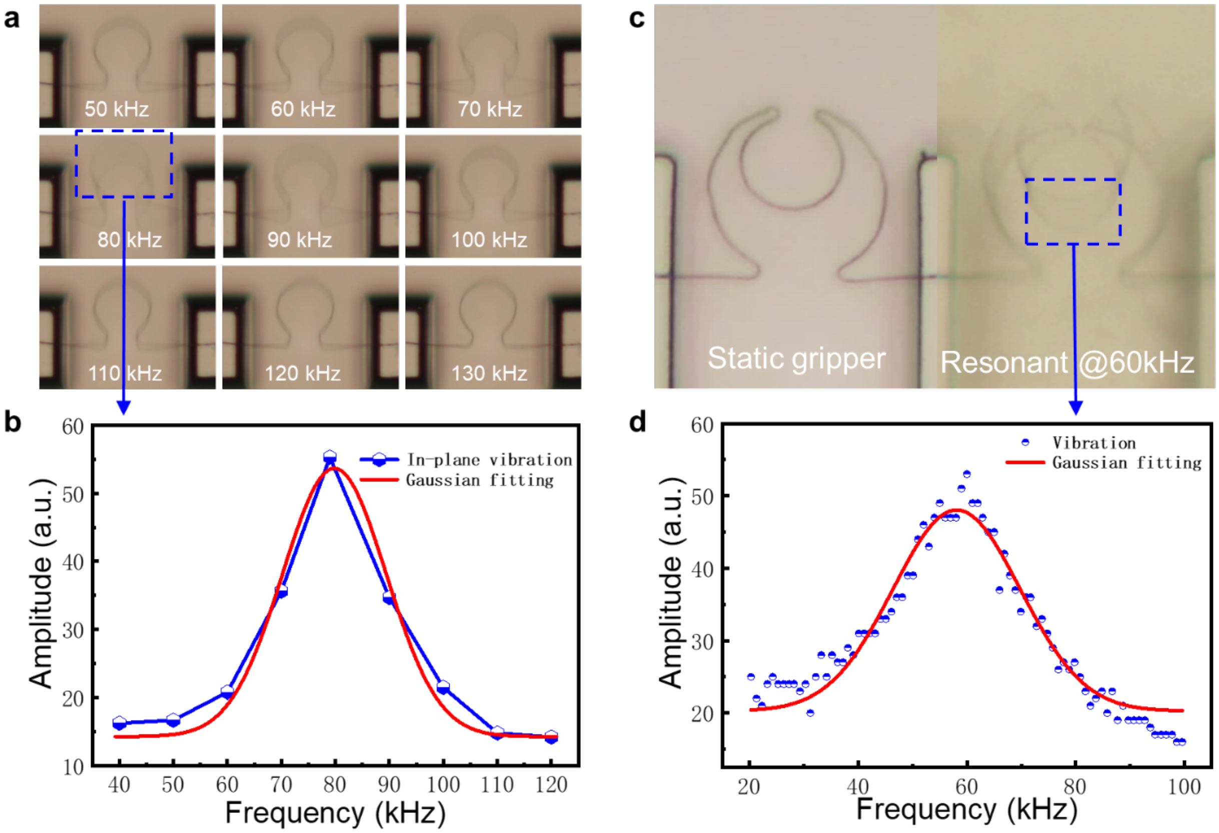

Supplementary Fig. 2. Vibrating Omega-ring and nested-ring (gripper) under different modulation frequencies. a Images of vibrating rings under excitation frequency of 50-130 KHz, where the vibration amplitude is extracted and plotted against modulation frequency in **b**, indicating a resonant frequency at 80 kHz, with a quality factor of 2.8. **c** Images of the grippers in static and resonant vibration, respectively, under excitation frequency of 60 kHz. **d** The extracted vibration amplitude plotted against varied frequency.

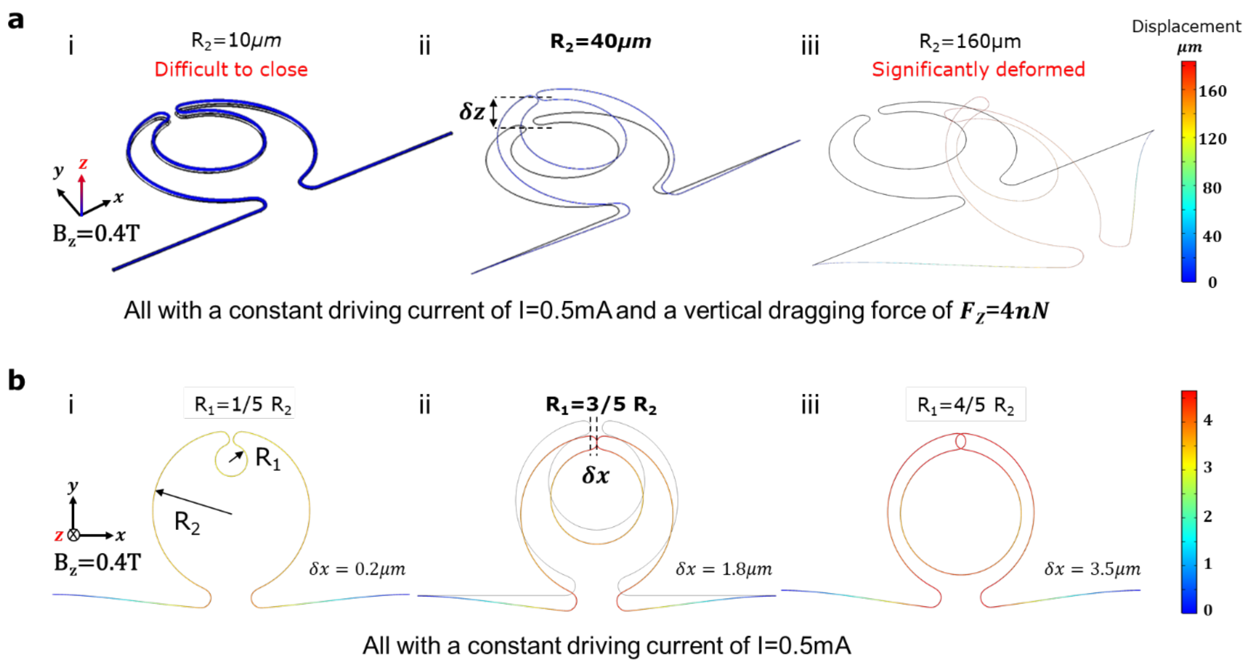

Supplementary Fig. 3 Deformation and structural stability of grippers of different size and ring designs. a Different deformations (or structural stabilities) of the grippers of different size, when subject to a constant vertical *z*-direction dragging force exerted at the tips. The smallest gripper has the best stability but difficult to sufficiently close with the same driving current of I=0.5 mA and $\mathbf{B}_z$ =0.4T; **b** A comparison of the gripping amplitude for the grippers with different inner ring sizes, under the same current bias conditions.

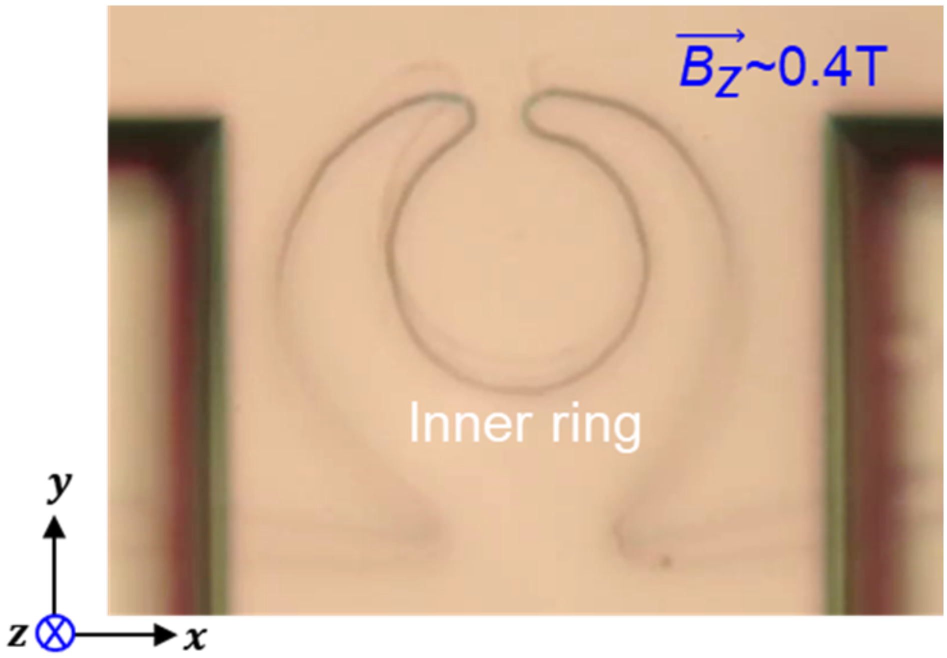

Supplementary Fig. 4 Out-of-plane deflection of the inner ring of a gripper structure. Overlapped optical images of a gripper in its initial status (defocused contour) and under a significant vectorial LF stress to contract (tend to close the tips, with I_{bias}>0.3 mA, $\mathbf{B}_z$=0.4T), where the inner ring opts to incline or flip out of the *x*-*y* working plane as a special means to release the accumulated stress in the gripper structure.

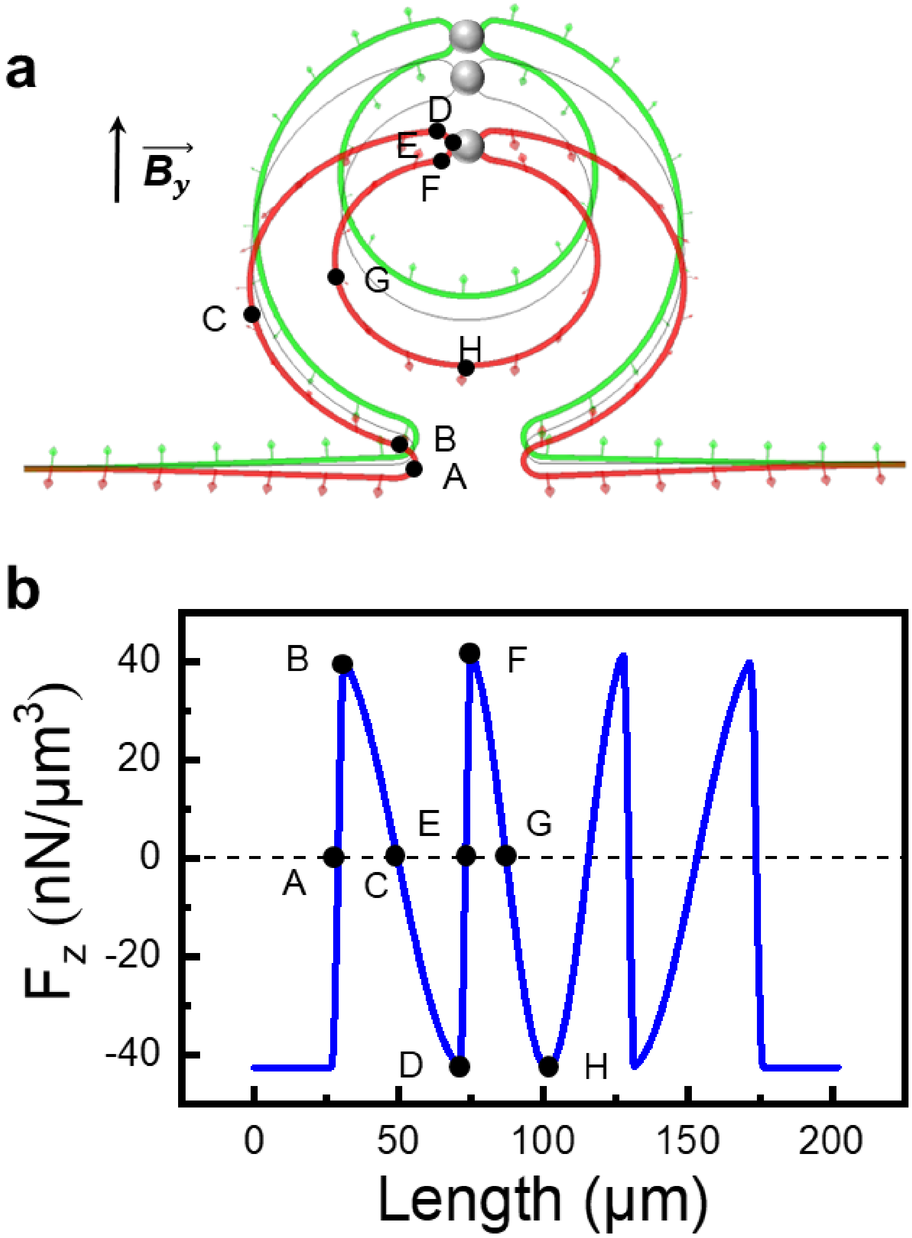

Supplementary Fig. 5 Finite element analysis of a bent gripper under LF driving.

a Within a static background magnetic field of $\overrightarrow{\mathbf{B_y}}$, the current-carrying NW segments will experience an upward (red line) or a downward (green line) Lorentz forces, resulting in an upward or downward bending, respectively. **b** the extracted upward-bending forces exerted on/along the gripper.

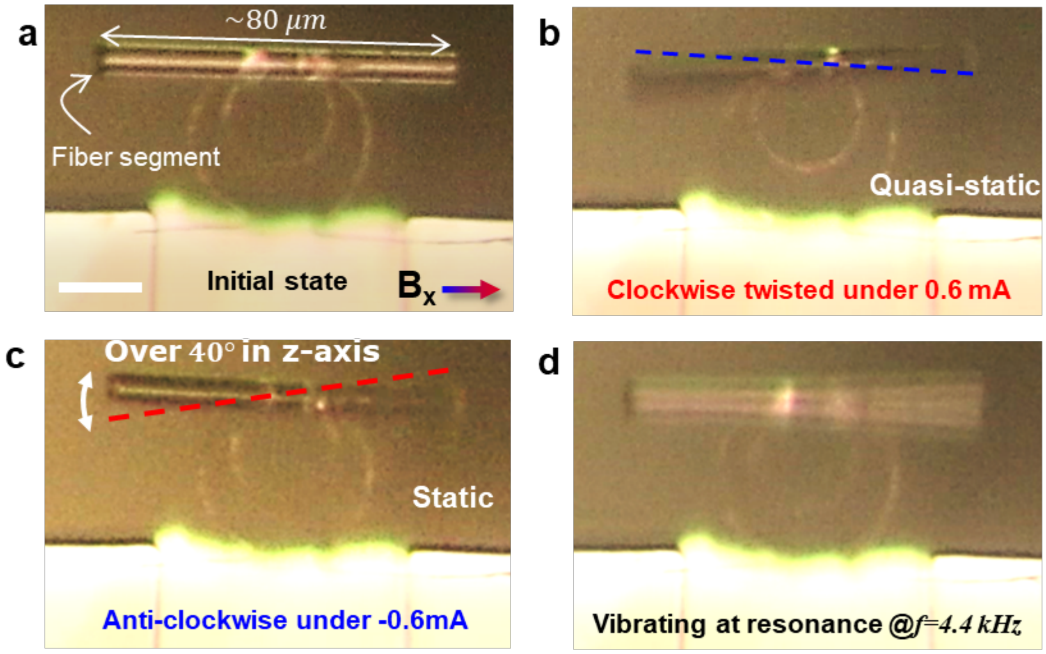

Supplementary Fig. 6 Twisting manipulation of gripper. a Suspended microscale fiber segment of ~80 μm long and ~7 μm wide held by a single gripper, **b-c** Quasi-static twisting over $\pm$ 20° under constant bias currents, **d** High frequency resonant twisting excited by alternating bias current at f = 4.4 kHz. Scale bar stands for 20 μm.

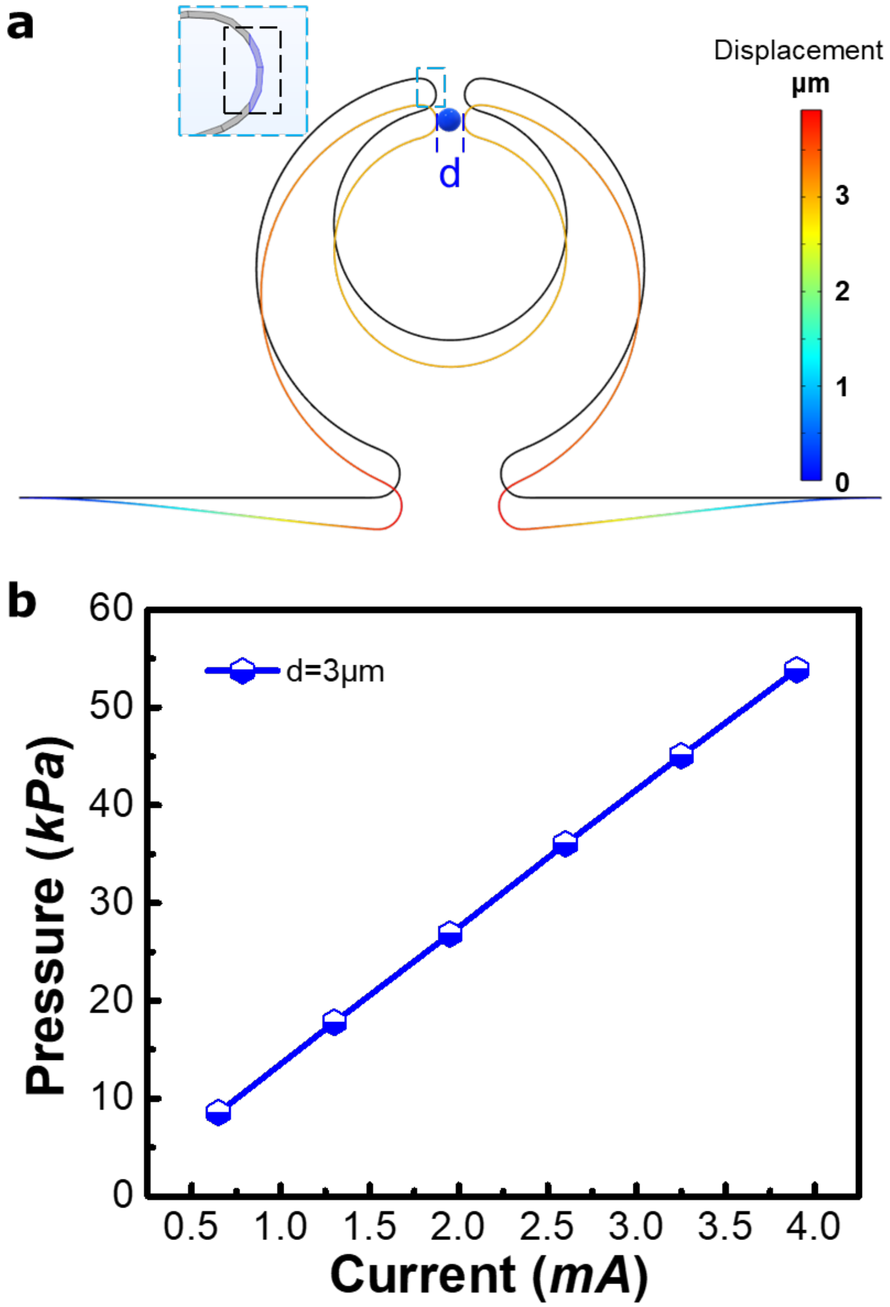

Supplementary Fig. 7 Finite element analysis of the clipping pressure exerted by the gripper tips, while holding a microsphere of 3 μm in diameter, which increases linearly, from 8.6 to 54kPa, while the driving current augmented from 0.6 to 4 mA. The color legend in **a** represents the absolute local displacement.

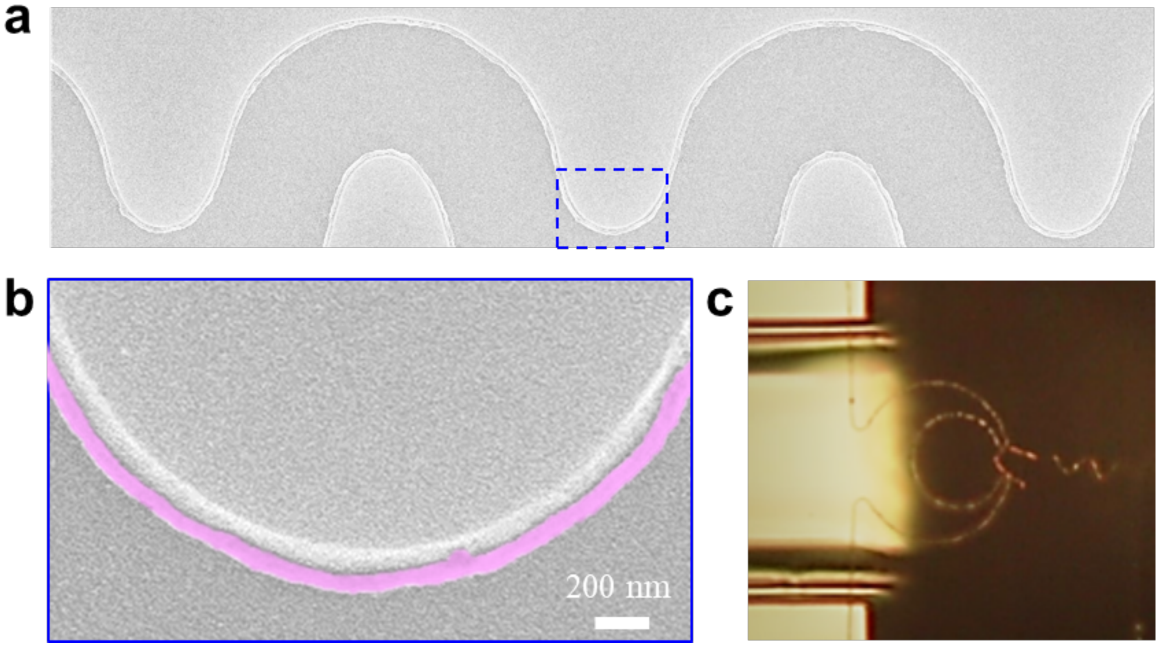

Supplementary Fig. 8 IPSLS guided growth of a SiNW spring for stretch force testing by gripper. a-b The SEM of images of a SiNW spring with a diameter of ~100 nm grown along the wavy guiding edges. **c** Holding of the SiNW spring by a gripper, where the tips are not touched to each other.

Supplementary Table 1 Comparison of SiNW grippers to the other finger-like grippers in the literature.

**Movable Materials**	**Fabrication**	**Driven by** (Volt. or Amp. or Magnetic)	**Gripper Operations**	**Open/Close** Response Time	**Action Parts** (Length×Width)	**Amplitude/Body length Ratio**	**Refs.**
Buried oxide layer in SOI	Focused Ion Beam	Electrostatic force (Voltage up to 200 V)	Hold and release	30 s	Movable arms (50 μm× 10μm)	0.32	1
Carbon nanotubes (CNT)	Manipulation Attaching	Electrostatic force (Voltage up to 8 V)	Hold but difficult to release	No data	50 nm CNT arms (4 μm× 1.5 μm)	0.25	2
Silicone mixed with carbon black	Blade-casting & Patterning	Electrostatic force, (Voltage up to 3.5 kV)	Hold and release	200 μs*	Deformable arms (114 mm× 47 mm)	0.4	3
Steel electrodes and PVC tapes	Patterning	Electrostatic force (Voltage up to 6-10 kV)	Unfold and fold	8 s * (Full range) 1 s * (partial range)	Deformable arms (>20 cm)	0.3	4
Parallel stack of Si nanowires (SiNWs)	Bosch etching + oxidation	Electrostatic force (Voltage up to 10 kV)	Hold and release	No data	SiNW arms (~100 nm) (70 μm × 20 μm)	0.29	5
Ecoflex filled with iron particles	Molding	Magnetic Force (Magnetic field 1.2 T)	Hold and release	25 s*	Movable arms (20 mm × 10 mm)	0.1	6
Polymer mixed with magnetic powders	Micro-molding	Magnetic Force (Magnetic field ~0.6 T)	Hold and release	<0.1 s*	Movable arms (1060 μm × 50 μm)	0.14	7
Stiff AFM probe with ferromagnetic beads	FIB milling	Electromagnetic (<40mT)	Hold and release	13 s	Deformable panels (50 μm × 100 μm)	0.17	8
Silicon with neodymium magnets	Lithography and etch	Magnetic Force (but no data)	Hold by using a pair of static arms	<0.2s *	Two microrobots (2 × 3 mm)	Static	9
Polymer arms with Cr/Au/Ni multilayers	Lithography and deposition	Thermal actuation (Temperature 20 ℃~37 ℃)	Only folding for once	7 min*	Deformable arms (300 μm × 200 μm)	0.67	10
SU8 with Cr/Au coating layer	Shadow mask and deposition	Thermal actuation (Voltages up to 2 V)	Hold and release	0.2 s* (full range) 0.03 s* (50%)	Movable arms (650 μm × 100 μm)	0.02	11
Pt and TiO_2 layers on polymeric panels	Photolithography	Electrochemical actuation (Voltage up to 0.5-1.1 V)	Flatten and curve	<0.1 s	Deformable arms (50 μm × 45 μm)	0.28	12
SiO/SiO_2 bilayer on patterned struct.	Photolithography	Stress actuation (dissolution in PBS)	Only folding for once	>24 hours to close*	Deformable arms (10 μm× 6 μm)	0.42	13
Single SiNWs with Ag coating	IPSLS growth engenering	Lorentz force (-0.2V~ 0.2 V, 0.4 T）	Multi-dimensional hold and release + resonant	< 10 μs* @ 60 kHz resonance	150 nm thick SiNW, morphed into grippers of (45 μm × 45 μm)	0.56	This work

*Values estimated from the plots and graphs in the references.

Supplementary References

[1] B. K. Chen, Y. Zhang, D. D. Perovic, Y. Sun, *J. Micromech. Microeng.* **2011**, *21*, 105004.

[2] P. Kim, C. M. Lieber, *Science* **1999**, *286*, 5447.

[3] J. Shintake, S. Rosset, B. Schubert, D. Floreano, H. Shea, *Adv. Mater*. **2016**, *28*, 231.

[4] M. Taghavi, T. Helps, J. Rossiter, *Sci. Robot*. **2018**, *3*, eaau9795.

[5] O. Ozsun, B. E. Alaca, Y. Leblebici, A. D. Yalcinkaya, I. Yildiz, M. Yilmaz, M. Zervas, *J. Microelectromech. S*. **2009**, *18*, 1335.

[6] S. Liu, S. Wang, S. Xuan, S. Zhang, X. Fan, H. Jiang, P. Song, X. Gong, *ACS Appl. Mater. Inter*. **2020**, *12*, 15675.

[7] E. Diller, M. Sitti, *Adv. Funct. Mater.* **2014**, *24*, 4397.

[8] H. Xie, X. Meng, H. Zhang, L. Sun, *IEEE T. Ind. Electron.* **2019**, *67*, 2065.

[9] L. Feng, P. Di, F. Arai, *Int. J. Robot. Res.* **2016**, *35*, 1445.

[10] E. Gultepe, J. S. Randhawa, S. Kadam, S. Yamanaka, F. M. Selaru, E. J. Shin, A. N. Kalloo, D. H. Gracias, *Adv. Mater.* **2013**, *25*, 514.

[11] N. Chronis, L.P. Lee, *J. Microelectromech. S*. **2005**, *14*, 857.

[12] Q. Liu, W. Wang, M. F. Reynolds, M. C. Cao, M. Z. Miskin, T. A. Arias, D. A. Muller, P. L. Mceuen, I. Cohen, *Sci. Robot*. **2021**, *6*, eabe6663.

[13] K. Malachowski, M. Jamal, Q. Jin, B. Polat, C. J. Morris, D. H. Gracias, *Nano Lett.* **2014**, *14*, 4164.
