# Supplementary material for: Ultracompact single-nanowire-morphed grippers driven by vectorial Lorentz forces for dexterous robotic manipulations
Source: Nat Commun. 2023 Jun 24;14:3786. doi: 10.1038/s41467-023-39524-z (PMC10290722; doi:10.1038/s41467-023-39524-z)
Supplement: Supplementary file 2 — Description of Additional Supplementary Files [file 41467_2023_39524_MOESM2_ESM.pdf]

<u>Description of Additional Supplementary Files</u>

Supplementary Movie 1 The vibration of a single Omega-ring at different modulation frequency (40-100 kHz).

Supplementary Movie 2 The video of microsphere picking from the edge of a platform by a LF-driven gripper.

Supplementary Movie 3 The twisting operation of a fiber segment by a gripper.

Supplementary Movie 4 High-frequency vibration assisted release of a microsphere on gripper under magnetic field along z-direction at 1.5 kHz.
